# Outbreak detection algorithms based on generalized linear model: a review with new practical examples

**DOI:** 10.1186/s12874-023-02050-z

**Published:** 2023-10-14

**Authors:** Bushra Zareie, Jalal Poorolajal, Amin Roshani, Manoochehr Karami

**Affiliations:** 1 Department of Epidemiology, School of Public Health, Hamadan University of Medical Sciences, Hamadan, Iran; 2 Department of Statistics, Lorestan University, Khorramabad, Iran; 3 Department of Epidemiology, School of Public Health and Safety, Shahid Beheshti University of Medical Sciences, Tehran, Iran

**Keywords:** Early aberration, GLMs with negative binomial, GLMs with Poisson, Outbreak algorithm, Statistical surveillance

## Abstract

Public health surveillance serves a crucial function within health systems, enabling the monitoring, early detection, and warning of infectious diseases. Recently, outbreak detection algorithms have gained significant importance across various surveillance systems, particularly in light of the COVID-19 pandemic. These algorithms are approached from both theoretical and practical perspectives. The theoretical aspect entails the development and introduction of novel statistical methods that capture the interest of statisticians. In contrast, the practical aspect involves designing outbreak detection systems and employing diverse methodologies for monitoring syndromes, thus drawing the attention of epidemiologists and health managers. Over the past three decades, considerable efforts have been made in the field of surveillance, resulting in valuable publications that introduce new statistical methods and compare their performance. The generalized linear model (GLM) family has undergone various advancements in comparison to other statistical methods and models. This study aims to present and describe GLM-based methods, providing a coherent comparison between them. Initially, a historical overview of outbreak detection algorithms based on the GLM family is provided, highlighting commonly used methods. Furthermore, real data from Measles and COVID-19 are utilized to demonstrate examples of these methods. This study will be useful for researchers in both theoretical and practical aspects of outbreak detection methods, enabling them to familiarize themselves with the key techniques within the GLM family and facilitate comparisons, particularly for those with limited mathematical expertise.

## Background

The process of ongoing, systematic collection, analysis, interpretation, and dissemination of data for public health purposes, aiming to reduce the morbidity and mortality of health-related events and promote public health, is referred to as surveillance [1]. One of the main goals of surveillance is timely outbreak detection, which enables prompt investigations and implementation of control measures. In recent years, the surveillance system in pursuit of this goal has experienced significant and rapid growth. This growth can be attributed to two underlying factors: increased concerns regarding large-scale bioterrorism attacks and heightened public awareness of emerging and re-emerging infections. These advances have led to the introduction of syndromic surveillance systems, increased databases, and the creation of automated outbreak detection systems to process data for large numbers of infections [2]. The effectiveness of syndromic surveillance or automated systems in early outbreak detection relies, to a certain extent, on the detection of statistical aberrations. A substantial increase in the number of suspected cases recorded by the surveillance system beyond the expected values and threshold levels established through statistical methods may indicate a statistically significant aberration. Such an aberration necessitates epidemiological investigation to determine whether it represents a potential outbreak or a spurious signal originating from the surveillance system [3]. The fundamental principles underlying all statistical methods employed to identify outbreaks, known as outbreak detection algorithms, involve identifying the time interval during which the number of recorded infection reports under surveillance exceeds the expected values. This identification process consists of two steps: calculating the expected number on the unit time (daily or weekly) and comparing the expected value with the observed value using a statistical test. Outbreak detection algorithms differ in their approaches to estimating the expected value, and they can be broadly categorized into two groups based on the type of expected value they utilize: algorithms that calculate the mean over a selected baseline period and algorithms that employ sample estimation [2].

The desire to improve outbreak detection algorithms for early detection of outbreaks has led to an increased development of statistical methods. Various classification methods are proposed for detection methods. For example, Unkel et al. (2012) classified these algorithms based on their approach (e.g., time series, statistical process control, and the GLM family) and their methods for obtaining thresholds (e.g., parametric, semi-parametric, non-parametric, and non-thresholding methods) [2]. Some articles categorize outbreak detection algorithms based on different and more general frameworks. Buckeridge et al. (2003) introduced a space–time analytical framework, and Faverjon and Berezowski (2018) categorized a conceptual framework for temporal algorithms in the syndromic surveillance system [4, 5]. Applying different outbreak detection algorithms and comparing these methods with one another has been done in several articles based on simulated and real data. Among these, we can refer to Bédubourget al. (2017) [6].

This paper reviews outbreak detection algorithms based on the GLM family. The rationale behind reviewing the GLM family is explained as the following. Generalized Linear Models are a class of statistical models that allow modeling the relationship between a response variable (such as the number of cases of a particular disease) and one or more predictor variables (such as age, gender, location, and time). GLMs extend linear regression models to handle non-normal distributions of the response variable, such as binary, count, or continuous data with non-constant variance. GLMs achieve this by specifying a link function that relates the expected value of the response variable to the linear combination of the predictor variables, such as a log-link function for count data. The selection of the link function depends on the characteristics of the response variable and the research question being addressed. GLMs also allow modeling the dispersion of the response variable using a variance function, which can be specified to accommodate different types of data. In summary, GLMs offer a flexible and robust framework for modeling disease incidence data and detecting potential outbreaks. GLM-based outbreak detection algorithms offer several advantages over other methods, which contribute to their emergence, development, and widespread use. GLM algorithms do not require parameter resetting, unlike time series and Cumulative Sum Control Chart (CUSUM) methods. Additionally, GLM models can be adjusted to account for overdispersion^1^ and seasonal trends, which are common in epidemiological data. These models can also utilize information on past outbreaks to estimate parameters and account for random effects. while GLM-based methods were used by Stroup et al. and Serfling et al. years ago, these methods have gained much more attention after the publication of Farrington's article in 1997 [7–9]. The main purpose of introducing the regression models is to provide flexible algorithms. These algorithms can account for main trends and noise in the data, adjust for these trends, and identify practical anomalies across a wide range of diseases and syndromes under surveillance. One of the notable characteristics of surveillance system data is the significant variation in the frequency distribution of cases per week or day. GLM models offer a viable solution for effectively addressing a broad range of diseases with varying frequency distributions, which are monitored by the surveillance system and reported to health organizations such as the Ministry of Health. For example, based on Farrington's idea, an outbreak detection algorithm should be robust enough to handle diseases with low and high prevalence. Salmonella, specifically the Typhimurium DT104 strain with a weekly frequency of less than 100, and rotavirus, with a weekly frequency of several hundred, are good examples of such diseases [9].

The increasing development of outbreak detection algorithms has led to their growing diversity, especially in algorithms based on the GLM family. Alongside the advantages of GLM-based methods, the diversity of these methods is another important reason for writing this review. Such diversity is not as common in other methods, and it can sometimes cause confusion among those interested in this field. The slight differences between these methods in statistical theory can sometimes lead to errors in practice. Another reason for choosing the GLM family of methods is its familiarity among researchers in public health and epidemiology. The GLM family is commonly used in the methodology of various studies. This familiarity makes researchers who want to work in the field of disease surveillance and outbreak detection algorithms more likely to learn and utilize these methods. As a point, generalized additive models (GAMs) are a semi-parametric extension of the GLMs that are also used in outbreak detection algorithms [10]. For more information on this topic, refer to [11] and [12]. The use of outbreak detection algorithms based on GAMs could offer unique benefits. However, since the studies in this field have not expanded enough, we focus on the GLM family in this paper. Additionally, the surveillance package in the R program is the most important tool in the implementation of outbreak detection algorithms, and various GLM-based methods are available in this package. A review paper on GLM-based algorithms can help researchers make better use of this useful application. Since the GLM family includes a large family of outbreak detection algorithms, our goal in this study is to provide an overview of the history and development of GLM-based methods, to help researchers use these methods and available statistical packages properly, and to give them a general understanding of these techniques. Despite progress in the field of outbreak detection methods, these methods are not yet widely used in many countries and are not familiar to public health authorities. An educational review that highlights differences or introduces frameworks can assist researchers in developing countries.

## Main text

### History

Outbreak detection algorithms based on the GLM family were introduced, inspired by Shewhart control charts (1931) [13]. Suppose that the variable ${Y}_{t}$ indicates the frequency of a disease under surveillance system at time t, with a normal distribution $N \left(\mu {,}{\sigma }^{2}\right)$. According to Shewhart control charts, an alarm will be declared at time t, when ${y}_{t}-\mu >\upkappa \sigma$, for a predetermined value of k. It should be noted that in the surveillance system, only the increased rates are considered, so κ ≥ 0 is assumed. The reason for such an increase can be the presence of a point source, such as contaminated food, which increases the number of cases. GLM-based outbreak detection methods were introduced to make three major changes to Shewhart control charts. These methods can accommodate non-normal distributions of the random variable. Mean and variance in the control of $\mu$ and ${\sigma }^{2}$ are dynamic parameters, and these parameters can be estimated from the historical baseline data [2]. One of the first studies on outbreak detection algorithms based on GLM dates back to Serfling et al. (1963), who proposed a parametric regression-based method known as the Serfling method to analyze the weekly mortality data of pneumonia and influenza in 108 cities in the United States. In this method, the errors were assumed to have a normal distribution with constant variance, and the data were modeled using a trigonometric function with a linear trend by

$$E\left({y}_{t}\right)=\mu +\alpha t+\sum_{i=1}^{r} \left\{{\beta }_{i}\mathrm{sin}\left({\omega }_{i}t\right)+{\gamma }_{i}\mathrm{cos}\left({\omega }_{i}t\right)\right\}{,}$$

where ${y}_{t}$ is the number of cases in week/month t, and the sine and cosine terms describe the seasonal change. Costagliola’s study in 1991 was based on the Serfling method and aimed to diagnose outbreaks of influenza-like syndromes and forecast the non-epidemic level for the next winter retrospectively from 1984 to 1988 in a surveillance system [14]. In this method, first, a subset of the entire data should be selected as the "training period". Then, data related to past outbreaks should be excluded from the training period. For example, 15% of the highest values can be removed. Finally, a regression equation based on the Serfling method should be fitted to predict the expected non-epidemic level. An upper threshold should also be considered to declare warnings of an outbreak detection algorithm based on this method by choosing the upper percentile of the predicted values. In this method, the parameters will be estimated using the mean square error method [8, 14]. One major drawback of this approach is determining epidemic periods or how much of the data observed in past outbreaks should be excluded when fitting the model. Another limitation is that fitting the regression model assuming a normal distribution of errors may be inappropriate if the data are overdispersed. In short, this method cannot be easily applied to a wide range of time series that show different characteristics, such as seasonal trends or the number of disease cases. However, this method is a simple and powerful approach for some well-known diseases. For example, Flahault et al. (1995) used this method to diagnose outbreaks of influenza-like syndrome or gastroenteritis [3, 15].

### Original farrington

Many surveillance system data are overdispersed, so a quasi-Poisson regression model was introduced by Farrington et al. (1996) and applied for early detection of outbreaks based on reports received at the Communicable Disease Surveillance Center (CDSC).

Let ${y}_{i}$ be the baseline count of a disease under the surveillance system corresponding to the baseline week ${t}_{i}$, independently distributed with mean ${\upmu }_{i}$ and variance ${\mathrm{\varphi \mu }}_{i}$. Considering a linear time trend in reporting disease frequency, the regression model is defined as

$$g\left({\mu }_{i}\right)=\alpha +\beta {t}_{i},$$

where $g$(.) denotes a link function, and all estimates are obtained using the quasi-likelihood method. For the following models, the link function that relates the mean response variable to the linear combination is assumed to be the logarithm function, except for the Jackson model where the link function is assumed to be the linear function ($g\left(x\right)=x$). It should be noted that the time unit used is weeks. In surveillance systems where early case detection is one of the objectives, the use of the monthly time unit is not recommended.

Trends are included in the regression model by fitting a linear time variable. This adjusted log-linear regression is very sensitive to overdispersion, as it can detect small increases in reporting of diseases with low incidence, as well as large increases in reporting of diseases with high incidence. There are several points in Farrington's (1996) study that we briefly mention here without going into detail. By plotting the average count of microorganisms per week against the variance, Farrington et al. (1996) showed that the Poisson distribution assumption is not valid in situations where the means are less than one. When means are greater than 10, the distribution will be asymptotically normal. Furthermore, Organisms with low incidence have a highly skewed distribution. Correction of skewness in situations where we have overdispersion, by transforming the data through changing the threshold, will lead to a reduction in false positive alarms.

For Poisson and negative binomial distributions, skewness correction by applying the power of 2/3 yields

$$F\left({y}_{0}\right)=O({\mu }_{0}).$$

and for large means, the Taylor series leads to a confidence interval $(L{,}U)$ with

$$\begin{array}{c}L={\hat{\mu }}_{0}\mathrm{max}\left\{{\left\{1-\frac{2}{3}{z}_{\alpha }{\left(\frac{\hat{\tau }}{{\hat{\mu }}_{0}}\right)}^{1/2}\right\}}^{3/2}{,}0\right\}{,}\\ U = \hat{\mu}_{0} \left\{1 + \frac{2}{3}z_{\alpha}\left(\frac{\hat{\tau }}{{\hat{\mu }}_{0}}\right)^{1/2}\right\}^{2/3}, \end{array}$$

where ${z}_{\alpha }$ is the 100(1- α)-percentile of the normal distribution and $\tau$ is

$$\tau = \phi + var \left(\hat{\mu}_{0}\right) /{\mu}_{0}.$$

Values greater than the upper threshold of U are flagged as a possible outbreak. For the Poisson distribution, the 2/3 transformation can lead to a symmetric distribution. Therefore, it provides more accurate thresholds. Another point in the Farrington method is that including all the data in the threshold calculation will lead to larger thresholds and reduce the sensitivity of alarms. Because the baseline data contains large values related to past outbreaks. Manual monitoring of the baseline data to identify outliers and remove them from calculations is impractical. Instead, a weighting method is used to reduce the effect of large values in the data. The weighting function is chosen based on assigning very small weights to values with large residuals. Note that weighting significantly reduces the effect of past outbreaks, but does not eliminate it. If the number of baseline outbreaks are higher, the effect remains larger after weighting (9). The Farrington method is implemented in R software and the "surveillance" package by Höhle in the following nine steps (16):

1. Fitting the initial model and initial estimation of mean and overdispersion.
2. Calculation of the weights omega (correction for past outbreaks)
3. Refitting of the model
4. Revised estimation of overdispersion
5. Rescaled model
6. Omitting the trend, if it is not significant
7. Repeating the whole procedure
8. Calculating the threshold value
9. Computing exceedance score

**Fig. 1:**
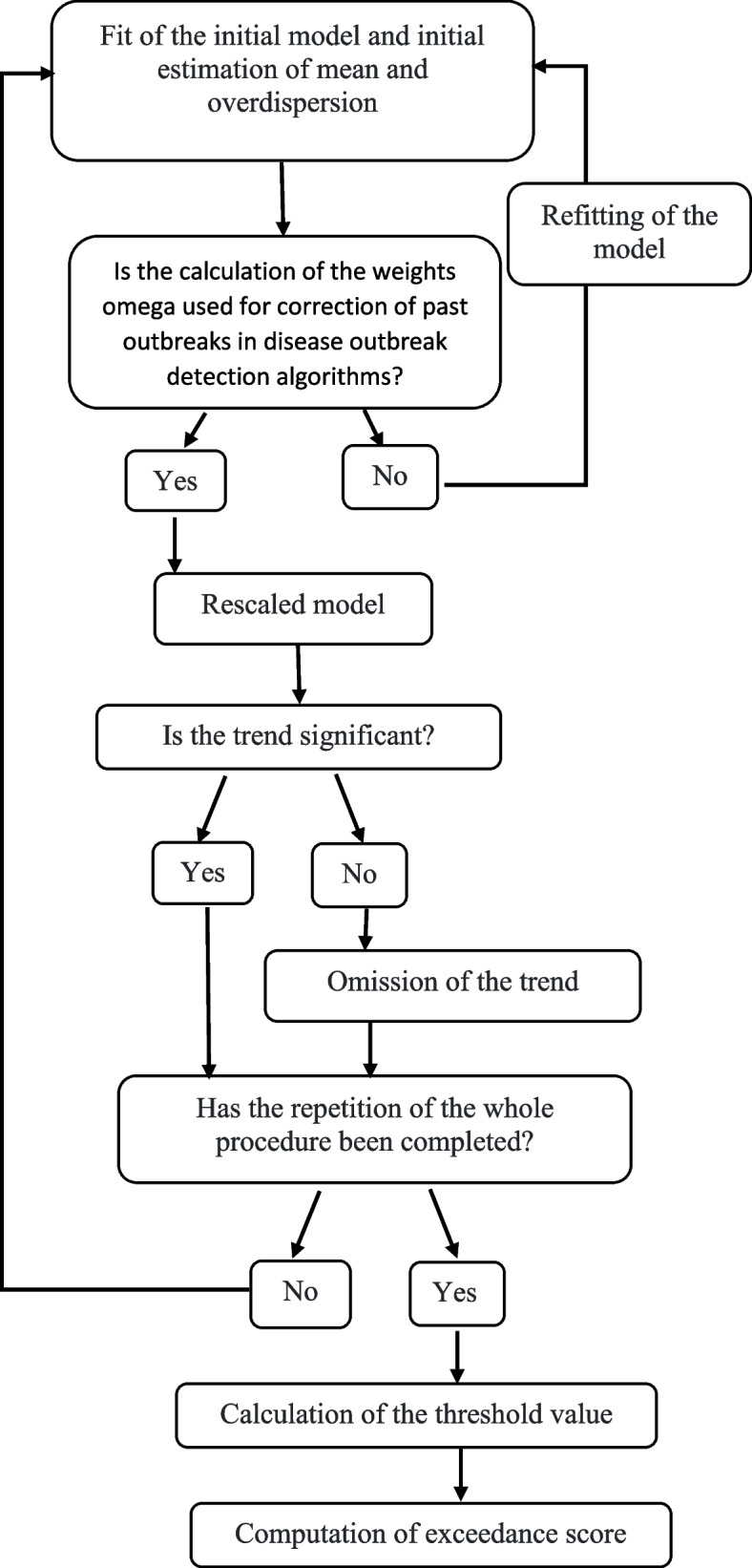
Farrington algorithm is implemented in R software, “Surveillance” package

The presented diagram is tailored to the nine steps outlined in the study. The Fig. 1, has been carefully designed to accurately represent each step and provide a clear visual aid to readers (Fig. 1).

### Farrington flexible

The improved method based on quasi-Poisson regression, developed by Noufaily et al. (2013), is described in this paper under the title Farrington Flexible. Another term used for the Farrington Flexible algorithm in some papers is "Improved Farrington". One of the limitations of the Farrington algorithm is the small number of baseline weeks used in the model formulation.

Due to the long-term changes in data collection and reporting in the system of surveillance, increasing the number of years to include more baseline data is not recommended. An alternative is to use more recent data and add seasonal variation to the Farrington model formulation. This model estimates the number of disease cases in the last week and includes a linear trend and a ten-level annual factor whose reference period includes comparable weeks in previous years. The corresponding linear log model is:

$$\mathrm{log}{\mu }_{i}=\theta +\beta {t}_{i}+{\delta }_{j\left({t}_{i}\right)}{,}$$

where $j({t}_{i})$ is the seasonal factor corresponding to week ${t}_{i}$. Assuming that $j\left({t}_{0}\right)=0$ and ${\delta }_{0}=0$, In this model, a trend is always included, regardless of its statistical significance, except when the data are highly sparse. The effect of past outbreaks is reduced through the Anscombe residuals, which are greater than 2.58.

This model is used to estimate excess deaths. The excess death approach evaluates the mortality burden of an epidemic. Various methods, including the Farrington algorithm, have been proposed to estimate excess deaths in heavily affected countries, such as during the COVID-19 pandemic [16, 17]. However, one of the main challenges in implementing this algorithm is when a small amount of data is available, especially for emerging diseases. Without sufficient data, the accuracy of estimates and forecasts can lead to inaccurate outbreak predictions [18]. Höhle has coded this method in the “surveillance” package, under the title Farrington Flexible. The implementation is illustrated in Salmon et al. (2016) [19, 20]. Farrington Flexible's algorithm is one of the algorithms used to monitor weekly data in Public Health England, such as Early Aberration Reporting System (EARS) which is a standard system at the United States CDC for conducting weekly syndromic surveillance [21]. The importance of early detection of outbreaks and ensuring the absence of incidents during mass gatherings are the main reasons why researchers are interested in using a daily monitoring surveillance system instead of weekly data, which has led to recent comparisons between algorithms for daily data [22–24]. It is important to specify the characteristics of each algorithm in these comparisons based on different criteria such as sensitivity, specificity, timeliness, etc. Noufaily et al. (2019) showed that Farrington Flexible algorithm has higher sensitivity and specificity in daily syndromic surveillance compared to methods such as EARS and the Rising Activity, Multilevel Mixed Effects, Indicator Emphasis (RAMMIE), which are used at Public Health England for syndromic daily surveillance [25].

### Some regression models mentioned in the articles

#### Jackson model

Jackson et al. (2007) developed a GLM model based on the Poisson distribution, using a three-year baseline data and Poisson errors. The effect of days of the week, month, linear time trend, and holiday variables are considered in this model. The model for the expected frequency on day t is:

$$\mathrm{E}\left({\mathrm{Y}}_{\mathrm{t}}\right)=\beta 0+\beta 1\text{ (Sunday) }+\dots +\beta 6\text{ (Friday) }+\beta 7\text{ (January)}$$

$$+\dots +\beta 17 (\mathrm{November}) +\beta 18 (\mathrm{Holiday}) +\beta 19 (\mathrm{time trend}).$$

If population data are available, binomial logistic models can be fitted in the same way as the Poisson distribution. The flexibility of the GLM approach allows more variables to be included in the model based on the researcher's opinion, such as random effects [26]. As we mentioned in the introduction, regression models can be tailored to different types of data and customized to meet the specific requirements of a given population or disease. However, using least-squares regression alone is not enough to eliminate systematic effects in epidemiological data. This is due to the fact that least-squares regression assumes residuals are independent and identically distributed with a normal distribution. In many cases, this assumption is violated in epidemiological data, where residuals are not independent or do not conform to a normal distribution. To address these issues, more advanced regression techniques are required. These techniques can account for the non-normality and non-independence of residuals and are essential for removing systematic effects from the data. Therefore, it is important to select the appropriate regression technique based on the nature of the data and the research question of interest [27, 28].

### Periodic poisson GLM method

Based on the simulated data, Bédubourg et al. (2017) fitted a Poisson and negative binomial regression model, where ${\mu }_{t}$ is defined as:

$$\mathrm{log}\left({\mu }_{t}\right)=\theta +\beta t+\sum_{j=1}^{m} \left({\gamma }_{1}\mathrm{cos}\left(\frac{2\pi jt}{52}\right)+{\gamma }_{2}\mathrm{sin}\left(\frac{2\pi jt}{52}\right)\right).$$

According to this model, the mean is equal to the predicted value at week t and the variance will be estimated in the negative binomial distribution through the model [6]. We have mentioned this method in Table 2 under the title of “Periodic Poisson GLM method”.

### Poisson regression charts based on generalized likelihood ratio (GLR)

The last method we explain in this article is the outbreak detection algorithm by Poisson regression charts based on generalized likelihood ratio (GLR). Höhle (2006) presented a seasonal Poisson regression chart and used time-varying and linear in-control mean. The new technique in Höhle's article was that the magnitude of the changes was not necessarily predetermined.

Let observations ${y}_{1}{,} {y}_{2}{,} {y}_{3}{,} \cdots$ follow a parametric distribution with density ${f}_{\theta }$. For the change point τ, the conditional density of ${y}_{t}|{x}_{t}{,} \tau$ define as follows

$${y}_{t}\mid {x}_{t}{,}\tau \sim \left\{\begin{array}{ll}{f}_{{\theta }_{0}}\left(\cdot \mid {x}_{t}\right)& \text{ for }t=1{,}\dots {,}\tau -1 (\text{in-control}) \\ {f}_{{\theta }_{1}}\left(\cdot \mid {x}_{t}\right)& \text{ for }t=\tau {,}\tau +1{,}\dots (\text{out-of-control}){,}\end{array}\right.$$

where ${X}_{t}$ represents the covariates known at time t, and ${f}_{{\theta }_{0}}$ and ${f}_{{\theta }_{1}}$ are Poisson probability density functions with means ${\mu }_{0{,}t}$ and ${\mu }_{1{,}t}$. The desired goal of the researcher is to determine τ in the shortest time; That is, new data will be collected until the researcher notices a change in the reported data. The stopping rule in sampling (in conditions other than the surveillance system) is when enough evidence is collected against the null hypothesis; ${H}_{0}:{\mu }_{t}={\mu }_{0{,}t}$. So, in the continuation of explaining mathematical calculations, don't forget that in order to determine τ from now on we should follow the calculations based on the stopping rule. The seasonal Poisson model for the in-control situation is considered as:

1 $$\mathrm{log}{\mu }_{0{,}t}={\beta }_{0}+\sum_{s=1}^{S} \left({\beta }_{2s-1}\mathrm{cos}(\omega st)+{\beta }_{2s}\mathrm{sin}(\omega st)\right).$$

In the above, ω = 2πT and T is a time period, for example for weekly data T = 52. The out-of-control state is also defined by an increase in the mean on a logarithmic scale as

2 $${\mu }_{1.t}={\mu }_{0.t}\cdot \mathrm{exp}\left(\kappa \right).$$

Hawkins et al. (2003) considered three different types of models in Eq. (1) based on whether or not the parameters are known, as follows:

1. All parameters of the model, i.e., (β, κ), are known.
2. The in-control parameters β are known, while θ = κ is unknown and has to be estimated during the surveillance system.
3. All parameters of the model, i.e., θ = (β, κ), are unknown and have to be estimated.

In the first approach, the uncertainty due to parameter estimation is ignored. The stopping rule based on the CUSUM method and the likelihood ratio in this approach is

3 $$N=inf\left\{n\ge 1:\underset{1\le k\le n}{\mathrm{max}} \left[\sum_{t=k}^{n} \mathrm{log}\left\{\frac{{f}_{{\theta }_{1}}\left({y}_{t}\mid {x}_{t}\right)}{{f}_{{\theta }_{0}}\left({y}_{t}\mid {x}_{t}\right)}\right\}\right]\ge {c}_{\gamma }\right\}$$

By not considering the covariate variables and with the minimum delay time and the average run length (ARL), γ, such that $E\left(N\right)=\gamma$ when $\uptau =\infty$, and knowing the parameters ${\theta }_{0}$ and ${\theta }_{1}$, the stopping rule [3] can be rewritten as

$$N=inf\left\{n: {l}_{n}>{c}_{\gamma }\right\}{,}$$

where

$${l}_{0}=0, {l}_{n}=max\left(0,{l}_{n-1}+\mathrm{log}\left\{\frac{{f}_{{\theta }_{1}}\left({y}_{n}\right)}{{f}_{{\theta }_{0}}\left({y}_{n}\right)}\right\}\right), n\ge 1.$$

Shu et al. (2004) showed how the uncertainty due to parameter estimation affects the ARL in different methods of outbreak detection algorithms, such as Shewhart control charts and the exponentially weighted moving average (EWMA) method. Therefore, there are shortcomings in using the first type. The third type is more realistic due to the characteristics of the surveillance or monitoring system. However, the theoretical and mathematical features of this type make modeling and parameter estimation more difficult. Lai (1995) showed that by considering the second approach and using the GLR method, Eq. (3) can be written as

4 $${N}_{G}=inf\left\{n\ge 1:\underset{1\le k\le n}{\mathrm{max}} \underset{\theta \in\Theta }{\mathrm{sup}} \left[\sum_{t=k}^{n} \mathrm{log}\left\{\frac{{f}_{\theta }\left({y}_{t}\mid {x}_{t}\right)}{{f}_{{\theta }_{0}}\left({y}_{t}\mid {x}_{t}\right)}\right\}\right]\ge {c}_{\gamma }\right\}$$

Höhle (2006) showed that by considering Eqs. (1) and (2) for the seasonal Poisson chart based on the GLR method in a recursive relationship, the ARL can be obtained. For more details, refer to Höhle’s paper (2006) [29–31]. This algorithm is available in the surveillance package in the R software under the titles “GLR Negative Binomial” and “GLR Poisson” [19].

### Comparing farrington, farrington flexible, jackson, periodic poisson GLM, and GLR algorithms for disease outbreak detection

Different outbreak detection algorithms have been evaluated and compared by a real or simulated dataset based on different measures [6, 32, 33]. The selection of measures for evaluating algorithm performance depends on the specific goals pursued by each surveillance system. In short, surveillance systems need to be optimized. This means that designers and users of monitoring systems should know which performance measures to consider and which algorithms perform well or poorly in different settings based on those measures. Using multiple datasets, including real data and simulated data, along with determining details related to past outbreaks such as outbreak size, is effective in facilitating better comparisons of algorithms [26].

We present the results of three studies that compare GLM-based algorithms with different settings. The study characteristics and comparison results are summarized in Table 1 [6, 25, 26], based on nine criteria which are introduced below.

- Specificity: The probability of no alarm given that an outbreak does not occur.
- Sensitivity: The probability of alarm given that an outbreak occurs.
- False Positive Rate (FPR): defined as the proportion of weeks corresponding to an alarm in the absence of an outbreak.
- Probability of Detection (POD): For each scenario and for each current week period, if an alarm is generated at least once between the start and the end of an outbreak, the outbreak is considered to be detected.
- Probability of Detection during the first week (POD1week): which makes it possible to evaluate the methods’ ability to enable early control measures.
- Positive Predictive Value (PPV): The probability that an alarm is truly an outbreak; True Positive/ (True Positive + False Positive).
- Negative Predictive Value (NPV): The probability of no outbreak given that no alarm is generated.; True Negative/ (True Negative + False Negative).
- *F*_1_: This measure defined as the harmonic mean of the sensitivity and the PPV: *F*_1_ = 2 × (*Sensitivity* × *PPV*)/( *Sensitivity* + *PPV*) [34].
- Timeliness: It is the proportion of days elapsed to detect an outbreak since its start.

**Table 1:** The characteristics and result of three studies that compared GLM-based algorithms

First Author, Year	Gabriel Bédubourg, 2017	Michael L Jackson, 2007	Angela Noufaily, 2019
Characteristics			
Simulated/ Real Dataset	Simulated	Real & Simulated	Simulated
Type of Surveillance System	Weekly Health Surveillance	Daily Syndromic Surveillance	Daily Syndromic Surveillance
Measures to Assess the Performance of the Algorithms	FPR^a^, POD^b^, POD1week^c^, Sensitivity, Specificity, PPV^d^, NPV^e^, F_1_	Sensitivity, Specificity, PPV, Timeliness	POD, Sensitivity, Specificity, PPV, Timeliness
Number of Algorithms Included	21	6	3
List of Algorithms Included	Farrington Flexible, Original Farrington, CDC (historical limits), CUSUM, CUSUM Rossi, CUSUM GLM, CUSUM, GLM Rossi, Bayes 1, Bayes 2, Bayes 3, RKI 1, RKI 2, RKI 3, GLR Negative Binomial, GLR Poisson, EARS C1, EARS C2, EARS C3 OutbreakP, periodic Poisson regression, periodic negative binomial regression	Three control chart-based algorithms commonly referred to as C1, C2, and C3, GLM, EWMA9, EWMA4	RAMMIE, EARS, Farrington Flexible
Result of Performance Measures	**Farrington Flexible**^**f**^**:** (1.0%, 99.0%, 43.3%, 34.0%, 20.5%, 95.0%, 58.3%, 0.30) **Original Farrington:** (2.3%, 97.7%, 56.9%, 45.5%, 29.0%, 95.4%, 45.0%, 0.35) **Periodic Poisson GLM:** (3.3%, 96.8%, 67.8%, 56.6%, 35.6%, 95.8%, 42.3%, 0.39) **GLR Poisson:** (15.15%, 84.5%, 75.5%, 60.3%, 45.9%, 95.9%, 16.4%, 0.24)	GLM model was more sensitive than the other algorithms and detected 54% (95% CI = 52%–56%) of the simulated epidemics when run at an alert rate of 0.01	Amongst the algorithm variants that have a high specificity (i.e. > 90%), Farrington Flexible has the highest sensitivity and specificity, but it is not most timely

^a^False Positive Rate

^b^Probability of Detection

^c^Probability of Detection during the first week

^d^Positive Predictive Value

^e^Negative Predictive Value

^f^The reported numbers are respectively (FPR, Specificity, POD, POD1week, Sensitivity, NPV, PPV and F1-measure)

In general, algorithms for detecting outbreaks, which belong to the same family of statistical methods, share similar functions. For example, both the Farrington and Farrington flexible algorithms detect outbreaks based on the deviation from the expected counts, taking into account seasonal and time trends in the data, and using a weighting scheme to downplay the effect of past outbreaks. The Farrington algorithm has less flexibility in choosing the baseline period and weighting, but requires fewer parameters for implementation. In contrast, the Farrington Flexible algorithm is more flexible in selecting the baseline period and weighting scheme and includes additional covariates to enhance its ability to detect outbreaks. These methods are used in various surveillance systems. For instance, the Farrington algorithm is integrated into the Second Generation Surveillance System (SGSS) in the UK to monitor infectious diseases and provide early warning of outbreaks to public health officials. Similarly, the Farrington Flexible algorithm used temperature and humidity as additional covariates to improve its accuracy in detecting outbreaks of dengue fever in Brazil, allowing public health officials to respond more promptly and effectively. Various studies conducted comparisons and evaluations of these two algorithms against different algorithms, using multiple indicators. Table 1 presents a subset of the results obtained from these studies [9, 18]. The Jackson model, as a GLM-based algorithm, did not receive as much attention in the literature as the previous two algorithms, and fewer comparative studies have been conducted on this method compared to other algorithms. However, similar to other GLM-based algorithms, this method has the capability to detect sudden epidemics more effectively and demonstrates better sensitivity in detecting outbreaks compared to widely used algorithms such as EWMA, particularly in the context of daily syndromic surveillance [5, 26]. The Periodic Poisson GLM method is a statistical technique that utilizes Poisson regression to model count data series. It considers the periodic nature of count data changes and models the controlled mean as a function of time and other covariates. By modeling the controlled mean at different time points, the method detects changes in the mean and provides an outbreak detection system. The method also considers seasonal and time trends in modeling the controlled mean and does not use a weighting scheme for observations. This makes the method particularly useful for diseases with a seasonal pattern [6]. The Generalized Likelihood Ratio (GLR) method is a statistical technique that is particularly well-suited for detecting sudden shifts in count data series. This method is found to be effective in detecting changes in controlled means of count data series, especially in the context of infectious diseases where counts tend to change periodically and seasonally. According to the study by Bédubourg, the periodic Poisson GLM method has a lower FPR, higher specificity, and higher F1-measure compared to the GLR Poisson method. Meanwhile, the GLR Poisson method shows better performance in terms of POD, POD1week, sensitivity, and NPV [6, 29].

### New examples of outbreak detection algorithms based on GLM for surveillance system

In this study, we illustrate the effectiveness of some of the introduced outbreak detection algorithms based on GLM using real-world data examples. The data used in these examples were obtained from the surveillance system of the Center for Disease Control and Prevention in Iran, covering the period from April 2016 to March 2022 for Measles and from February 2020 to July 2022 for Covid-19. Providing a brief explanation of the types of data, data preprocessing, and surveillance workflow involved in disease surveillance in Iran is necessary. In disease surveillance systems, data falls into three categories: proportional, count, and continuous. Count data is the most common type, such as the number of flu cases in the past week. Proportional data such as the percentage of positive Covid-19 cases among new samples taken on the day of sampling for Influenza-Like Illness (ILI), Severe Acute Respiratory Infection (SARI), and Acute Respiratory Infection (ARI). Disease surveillance systems classify reported cases into suspected, probable, and laboratory-confirmed cases and take appropriate actions based on the category. Count data for both Measles and Covid-19 were obtained from the routine or traditional surveillance system. The count data for both diseases were based on laboratory-confirmed cases, and for Covid-19, it included the number of hospitalized individuals. An expert evaluates and corrects the data for each disease to ensure the accuracy and completeness of patient variables, as well as to identify and remove any outliers or incorrect data.

**Fig. 2:**
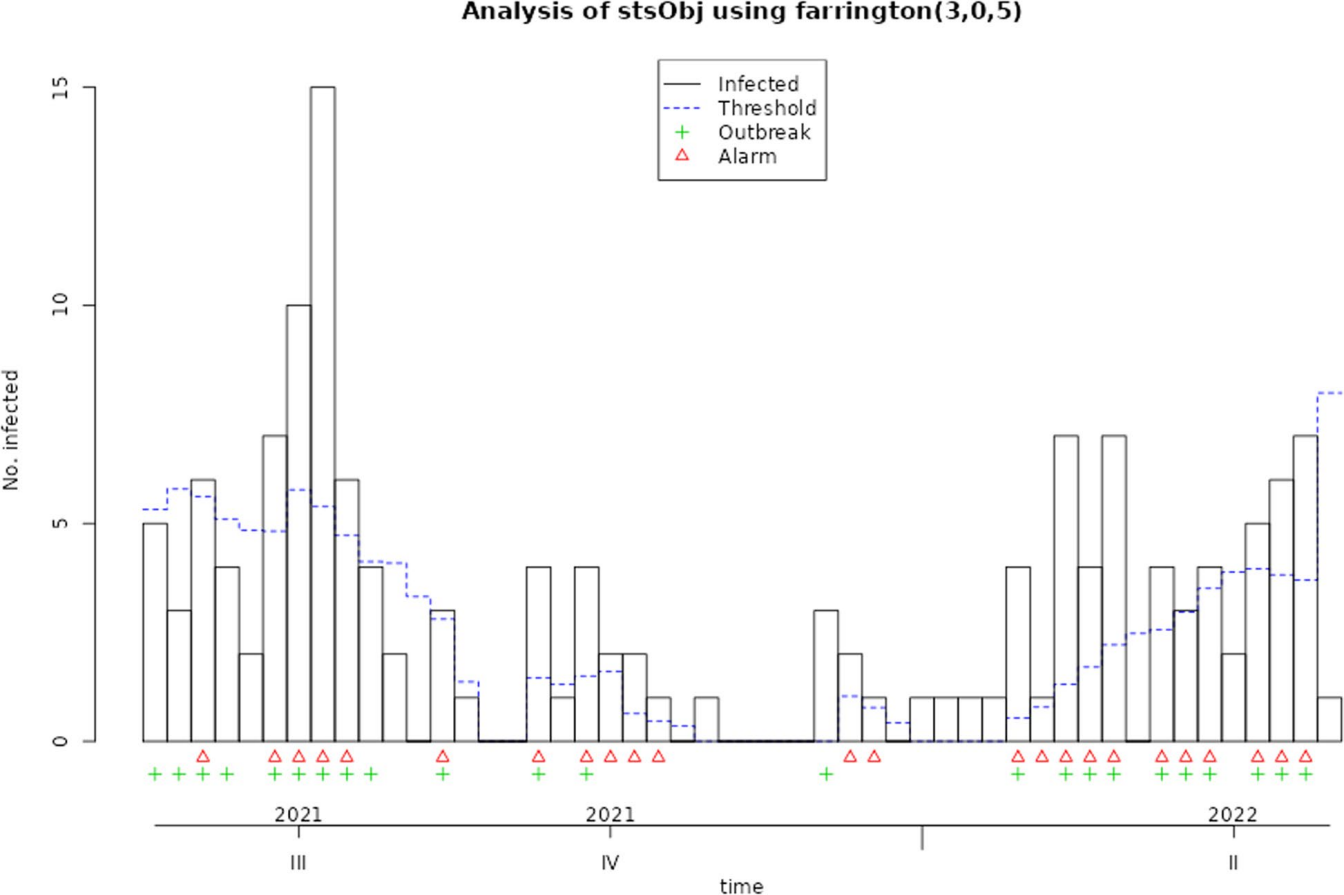
Applying the original farrington algorithm to measles data from 2018 to 2022

**Fig. 3:**
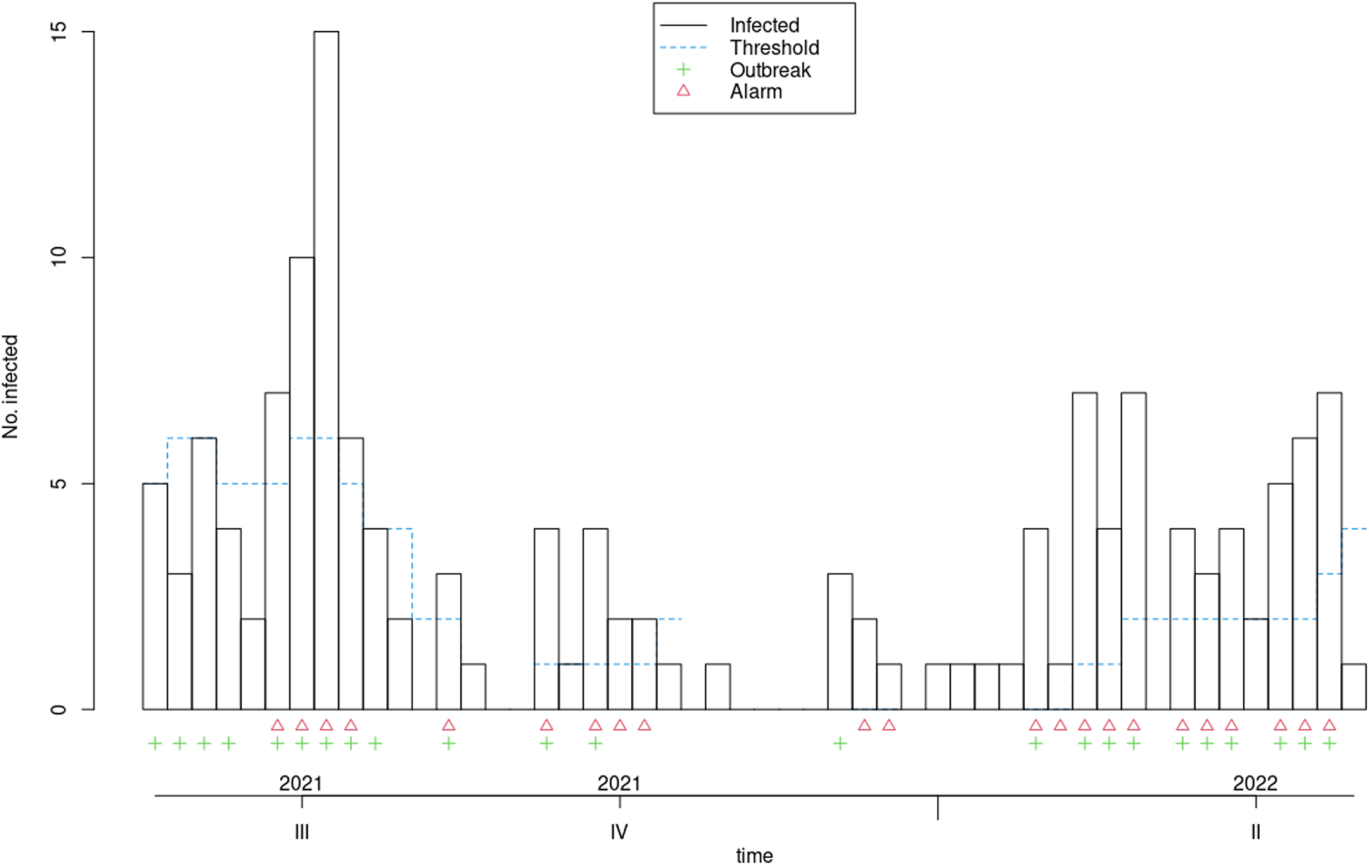
Applying the farrington flexible algorithm to measles data from 2018 to 2022

**Fig. 4:**
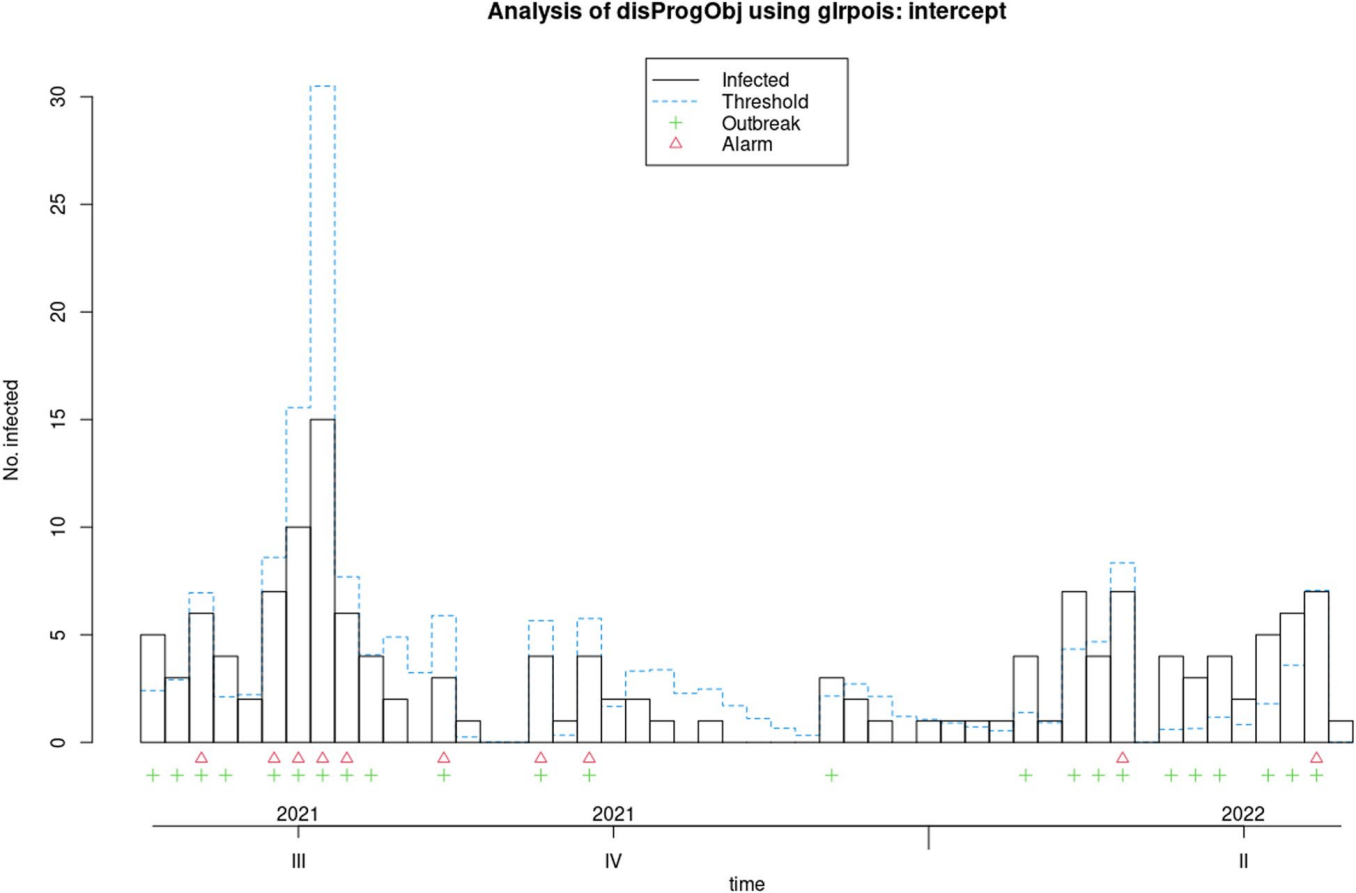
Applying the GLR poisson algorithm to measles data from 2018 to 2022

**Fig. 5:**
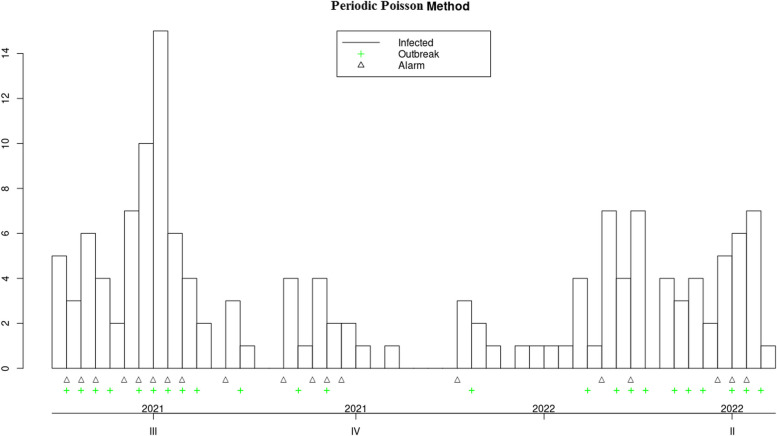
Applying the periodic poisson GLM algorithm to measles data from 2018 to 2022

**Fig. 6:**
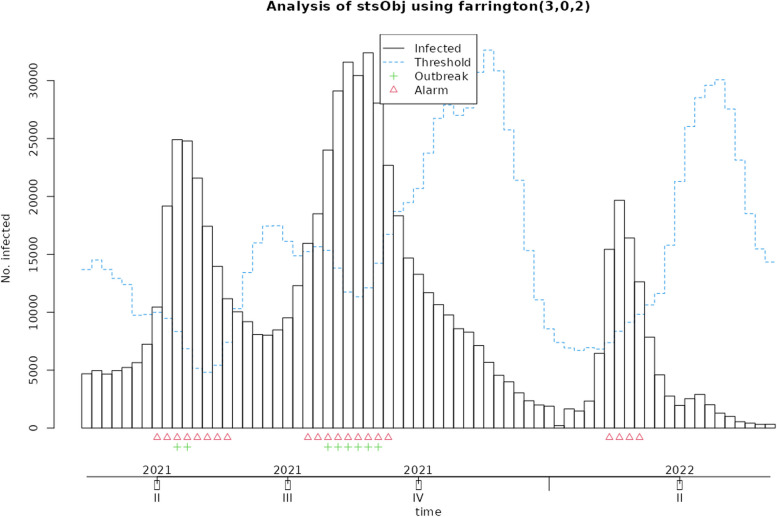
Applying the original farrington algorithm to Covid-19 data from 2020 to 2022

**Fig. 7:**
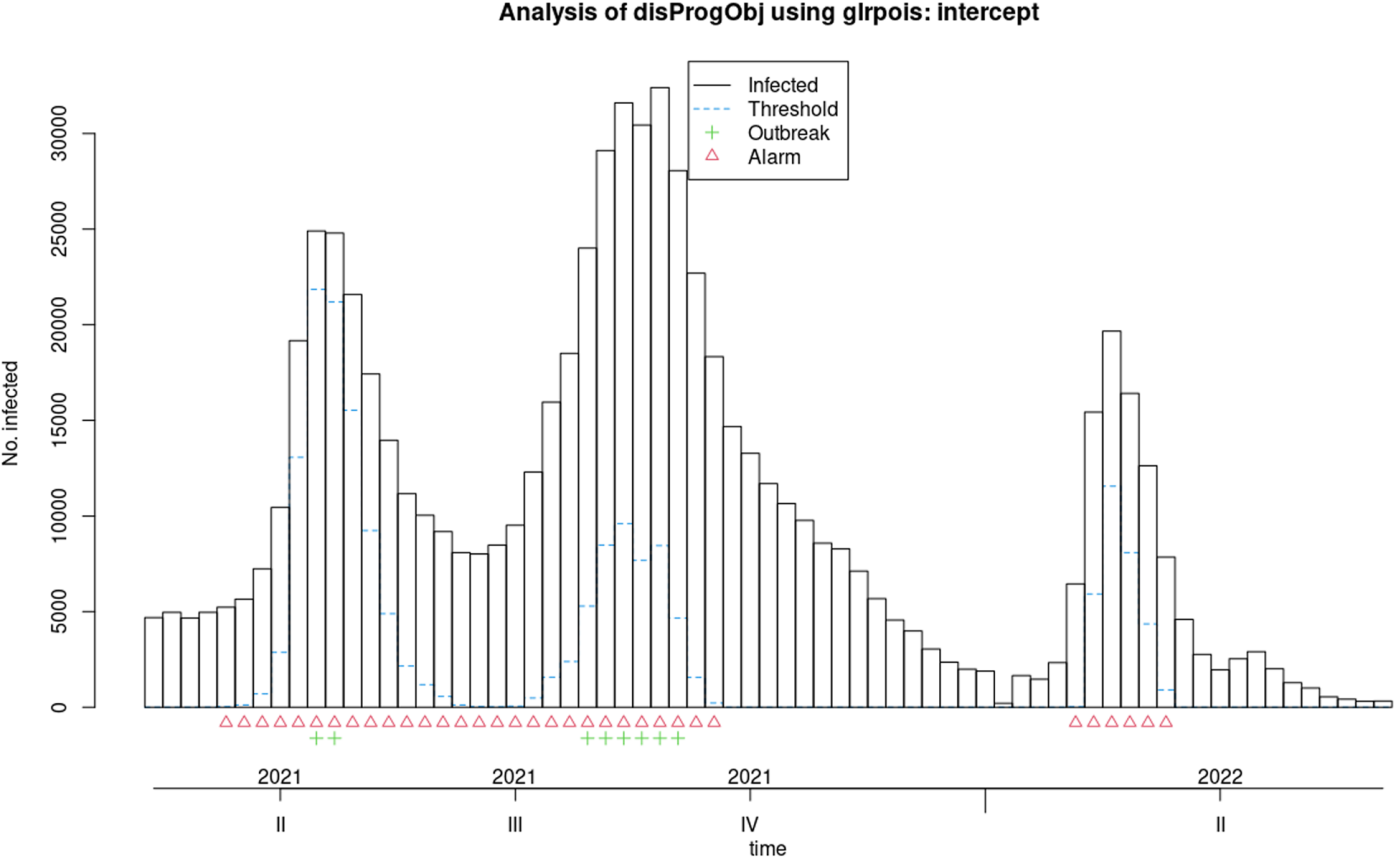
Applying the GLR poisson algorithm to Covid-19 data from 2020 to 2022

The disease surveillance system in Iran is implemented using the syndromic and routine or traditional surveillance approach. In this structure, healthcare providers and other stakeholders passively report notifiable diseases to higher levels of primary healthcare centers. The reports are then transferred to higher levels of the network system, where they are analyzed and used to identify potential outbreaks and inform public health interventions. This system relies on the cooperation and coordination of healthcare providers at all levels to ensure the timely and accurate reporting of disease cases. We selected two diseases with different incidence rates: measles, which has a low incidence rate, and Covid-19, an emerging disease with a short baseline history and high incidence rate. To detect outbreaks, we employed four methods: Original Farrington, Farrington Flexible, GLR Poisson, and Periodic Poisson GLM (Figs. 2, 3, 4, 5, 6, and 7). We collected data based on date, frequency, and status, and used this data to run outbreak detection algorithms.

In the 'Status' variable, we record the researcher's opinion as a binary variable based on whether an outbreak has occurred during that time period. For Measles, an outbreak was defined as a frequency of three or more, while for Covid-19, a frequency greater than three times the standard deviation was considered an outbreak. The researcher's opinion is indicated by a green marker in the graphs and is used to determine the performance of each algorithm, including calculating sensitivity and specificity. This opinion may change based on new guidelines and is specific to each disease. Data for both diseases were monitored on a weekly basis. These examples are for educational purposes only and were not used for predictive modeling. To use any algorithm effectively, it is advisable to select parameters based on more reliable evidence. Table 2 shows the software package used and the parameter details.

**Table 2:** Characteristics of GLM-based outbreak detection algorithms in examples

Method	Package’s name; Command	Range^a^(week)	Control parameters	Data	Reference to R packag and control parameters
Original Farrington	R package surveillance; algo.farrington()	270–319	b = 5, w = 3, weight, reweight = TRUE α = 0.05	Measles	[9, 19]
		52–120	b = 2, w = 3, weight, reweight = TRUE α = 0.05	Covid-19	
Farrington Flexible	R package surveillance; farringtonFlexible()	270–319	b = 5, w = 3, weight threshold = 2.58, thresholdMethod = “nbPlugin”, α = 0.05	Measles	[18, 19]
GLR Poisson	R package surveillance; algo.glrpois()	270–319	ARL = 5, dir = “inc”	Measles	[19, 29]
		52–120		Covid-19	
Periodic Poisson GLM Method	-	270–319	m = 2	Measles	[26]

^a^determines the desired time points which should be evaluated

### Evaluation the performance of GLM-based algorithms for detecting measles and Covid-19 outbreak

In this section, the performance of Original Farrington, Farrington Flexible, GLR Poisson, and Periodic Poisson algorithms in detecting outbreak of measles and Covid-19 diseases is examined based on various evaluation indicators. various indicators, including sensitivity, specificity, PPV, NPV, and FPR, which are presented in Table 3 for each algorithm.

**Table 3:** Comparison of the performance of epidemic detection algorithms for measles and COVID-19 based on performance evaluation indicators

Algorithms	Disease	Sensitivity	Specificity	Positive Predictive Value	Negative Predictive Value	False Positive Rate	Figures
Original Farrington	Measles	0.78	0.78	0.75	0.81	0.22	Fig. 2
Farrington Flexible	Measles	0.74	0.82	0.77	0.79	0.19	Fig. 3
GLR^a^ Poisson	Measles	0.44	1.00	1.00	0.68	0.00	Fig. 4
Periodic Poisson	Measles	0.48	0.70	0.58	0.61	0.30	Fig. 5
Original Farrington	Covid-19	1.00	0.79	0.38	1.00	0.21	Fig. 6
GLR Poisson	Covid-19	1.00	0.57	0.24	1.00	0.43	Fig. 7

^a^Generalized likelihood ratio

For measles outbreaks, both the Original Farrington and Farrington Flexible algorithms exhibit similar performance in terms of sensitivity and specificity, with the Original Farrington algorithm having slightly higher sensitivity and the Farrington Flexible algorithm having slightly higher specificity. Both algorithms are better at ruling out non-outbreak situations than identifying true outbreaks.

The GLR Poisson algorithm performs well compared to other algorithms based on some of its key performance indicators, such as specificity and PPV and FPR. Although it has lower sensitivity compared to algorithms like Farrington, it has a very high specificity and PPV, indicating that the cases it identifies as positive are very likely to be true positives.

Additionally, the FPR for this algorithm is zero, meaning it has no false positives. Overall, the GLR Poisson algorithm can be a useful algorithm in identifying disease outbreaks in real-time, particularly in situations where high specificity and PPV are crucial, such as in public health emergency response. The Periodic Poisson algorithm uses a periodic Poisson distribution model and can work well in identifying diseases with periodic patterns (such as influenza). In this algorithm, both sensitivity and specificity are lower than other algorithms, indicating that its performance in identifying anomalous data points is lower. The PPV and NPV are also lower than other algorithms. This algorithm has a lower specificity and performs better in identifying days with epidemics or outbreaks compared to identifying days with epidemics or outbreaks. The FPR for this algorithm is relatively high. The Original Farrington algorithm has better overall performance than the GLR Poisson algorithm for detecting Covid-19 epidemics, with higher specificity and lower false positive rate. However, the GLR Poisson algorithm may still be useful in certain situations where high sensitivity is required. It is worth noting that the performance of the GLR algorithm varies between detecting Covid-19 and measles.

In general, the choice of algorithm depending on the specific research or application requirements, as well as the characteristics of the data being analyzed. A careful evaluation of multiple algorithms may be necessary to determine the most appropriate one for a particular situation. Therefore, depending on the research needs and goals, a trade-off may be necessary to be made between sensitivity and specificity when choosing which algorithm to use.

## Conclusions

In recent years and with the pandemic of emerging infectious diseases, more researchers are interested in the development of outbreak detection algorithms and the correct practical use of these algorithms in the field of communicable disease management. Since numerous studies have been conducted in this field, conducting review studies in different classifications will contribute to the creation of better frameworks and the development of the theoretical field, as well as the practical use of the introduced algorithms. To provide better guidance for researchers in the field of outbreak detection methods, it is necessary to refine general reviews with broad questions into more specific reviews with focused questions. By reading review articles, researchers can gain a deeper understanding of the application and progress of these algorithms. This study focuses on the emergence and development of outbreak detection algorithms based on GLM, aiming to provide a comprehensive view of these algorithms for students of epidemiology, biostatistics, and health managers studying in the field of surveillance and outbreak detection algorithms.

## Data Availability

Not applicable.

## Abbreviations

GLM: Generalized Linear Model
CDSC: Communicable Disease Surveillance Center
GLR: Generalized Likelihood Ratio
CUSUM: Cumulative Sum Control Chart
EWMA: Exponentially Weighted Moving Average
ARL: Average Run Length
RAMMIE: Rising Activity, Multilevel Mixed Effects, Indicator Emphasis
EARS: Early Aberration Reporting System
FPR: False Positive Rate
POD: Probability of Detection
POD1week: Probability of Detection during the first week
PPV: Positive Predictive Value
NPV: Negative Predictive Value

## Footnotes

1 Overdispersion is a situation where the observed data show more variation than what would be expected under the statistical models. It can lead to biased estimates and less reliable significance tests, and can be caused by various factors such as unobserved heterogeneity or measurement error. Some models such as quasi-Poisson, mixed Poisson, or zero-inflated Poisson regression can be used to account for overdispersion.

## References

[1] Buchler J, Hopkins R, Overhage J, Sosin D, Tong V (2004). Framework for evaluating public health surveillance systems for early detection of outbreak recommendations from the CDC Working Group. MMWR Recomm REP.

[2] Unkel S, Farrington CP, Garthwaite PH, Robertson C, Andrews N (2012). Statistical methods for the prospective detection of infectious disease outbreaks: a review. J R Stat Soc A Stat Soc.

[3] Lawson AB, Kleinman K. Spatial and syndromic surveillance for public health: John Wiley & Sons; 2005.

[4] Buckeridge DL, Musen MA, Switzer P, Crubézy M, editors. An analytic framework for space–time aberrancy detection in public health surveillance data. AMIA Annual Symposium Proceedings; 2003: American Medical Informatics Association. PMC1479968 14728146

[5] Faverjon C, Berezowski J (2018). Choosing the best algorithm for event detection based on the intended application: a conceptual framework for syndromic surveillance. J Biomed Inform.

[6] Bédubourg G, Le Strat Y (2017). Evaluation and comparison of statistical methods for early temporal detection of outbreaks: a simulation-based study. PLoS ONE.

[7] Stroup DF, Williamson GD, Herndon JL, Karon JM (1989). Detection of aberrations in the occurrence of notifiable diseases surveillance data. Stat Med.

[8] Serfling RE (1963). Methods for current statistical analysis of excess pneumonia-influenza deaths. Public Health Rep.

[9] Farrington C, Andrews NJ, Beale A, Catchpole M (1996). A statistical algorithm for the early detection of outbreaks of infectious disease. J R Stat Soc A Stat Soc.

[10] Jones K, Almond S. Moving out of the linear rut: the possibilities of generalized additive models. Trans Inst Br Geograph.1992:434–47.

[11] Alimadad A, Salibian-Barrera M (2011). An outlier-robust fit for generalized additive models with applications to disease outbreak detection. J Am Stat Assoc.

[12] Chen P, Fu X, Ma S, Xu HY, Zhang W, Xiao G (2020). Early dengue outbreak detection modeling based on dengue incidences in Singapore during 2012 to 2017. Stat Med.

[13] Shewhart WA. Economic control of quality of manufactured product: Macmillan And Co Ltd, London; 1931.

[14] Costagliola D, Flahault A, Galinec D, Garnerin P, Menares J, Valleron A-J (1991). A routine tool for detection and assessment of epidemics of influenza-like syndromes in France. Am J Public Health.

[15] Flahault A, Garnerin P, Chauvin P, Farran N, Saidi Y, Diaz C (1995). Sentinelle traces of an epidemic of acute gastroenteritis in France. Lancet (British edition).

[16] Burki T (2020). England and Wales see 20 000 excess deaths in care homes. The Lancet.

[17] Yoneoka D, Kawashima T, Makiyama K, Tanoue Y, Nomura S, Eguchi A (2021). Geographically weighted generalized Farrington algorithm for rapid outbreak detection over short data accumulation periods. Stat Med.

[18] Noufaily A, Enki DG, Farrington P, Garthwaite P, Andrews N, Charlett A (2013). An improved algorithm for outbreak detection in multiple surveillance systems. Stat Med.

[19] Hoehle M, Meyer S, Paul M, Held L, Burkom H, Correa T. Temporal and Spatio-Temporal Modeling and Monitoring of Epidemic Phenomena. Surveillance R package version. 2017;1(0).

[20] Maëlle S, Dirk S, Michael H. Monitoring count time series in R: Aberration detection in public health surveillance. arXiv preprint arXiv:14111292. 2014.

[21] Fricker RD, Hegler BL, Dunfee DA (2008). Comparing syndromic surveillance detection methods: EARS'versus a CUSUM-based methodology. Stat Med.

[22] Abat C, Chaudet H, Rolain J-M, Colson P, Raoult D (2016). Traditional and syndromic surveillance of infectious diseases and pathogens. Int J Infect Dis.

[23] Mathes RW, Lall R, Levin-Rector A, Sell J, Paladini M, Konty KJ (2017). Evaluating and implementing temporal, spatial, and spatio-temporal methods for outbreak detection in a local syndromic surveillance system. PLoS ONE.

[24] Vial F, Wei W, Held L (2016). Methodological challenges to multivariate syndromic surveillance: a case study using Swiss animal health data. BMC Vet Res.

[25] Noufaily A, Morbey RA, Colón-González FJ, Elliot AJ, Smith GE, Lake IR (2019). Comparison of statistical algorithms for daily syndromic surveillance aberration detection. Bioinformatics.

[26] Jackson ML, Baer A, Painter I, Duchin J (2007). A simulation study comparing aberration detection algorithms for syndromic surveillance. BMC Med Inform Decis Mak.

[27] McCulloch CE, Searle SR. Generalized, linear, and mixed models: John Wiley & Sons; 2004.

[28] Pek J, Wong O, Wong AC (2018). How to address non-normality: a taxonomy of approaches, reviewed, and illustrated. Front Psychol.

[29] Höhle M. Poisson regression charts for the monitoring of surveillance time series. Discussion Paper, 2006.

[30] Shu L, Tsung F, Tsui K-L (2004). Run-length performance of regression control charts with estimated parameters. J Qual Technol.

[31] Lai TL (1995). Sequential changepoint detection in quality control and dynamical systems. J Roy Stat Soc: Ser B (Methodol).

[32] Kleinman K, Lazarus R, Platt R (2004). A generalized linear mixed models approach for detecting incident clusters of disease in small areas, with an application to biological terrorism. Am J Epidemiol.

[33] Miller B, Kassenborg H, Dunsmuir W, Griffith J, Hadidi M, Nordin JD (2004). Syndromic surveillance for influenzalike illness in ambulatory care setting. Emerg Infect Dis.

[34] Hripcsak G, Rothschild AS (2005). Agreement, the f-measure, and reliability in information retrieval. J Am Med Inform Assoc.

